# Similar Stressors Different Expression: Ethnic Disparities in Temporomandibular Disorders and Bruxism During Wartime

**DOI:** 10.1111/joor.70205

**Published:** 2026-04-17

**Authors:** Alona Emodi‐Perlman, Khalil Masarwe, Nour Mahajne, Lihi Keren, Ilana Eli

**Affiliations:** ^1^ Department of Oral Rehabilitation, The Maurice and Gabriela Goldschleger School of Dental Medicine, Grey Faculty of Medicine and Health Sciences Tel Aviv University Tel Aviv Israel

**Keywords:** armed conflict, bruxism, ethnicity, psychological, resilience, temporomandibular disorders

## Abstract

**Background:**

War, an intense and enduring source of stress, exerts wide‐ranging impacts on psychological well‐being, provoking significant stress and anxiety reactions even in individuals not directly exposed to combat.

**Objective:**

To examine how ethnicity influences bruxism and temporomandibular disorders (TMD) during an armed conflict.

**Methods:**

In a cross‐sectional design, 895 subjects from two ethnic origins (595 Jews and 301 Arabs) were evaluated through an online survey. The assessment tools covered demographic data, TMD screening, subject‐based sleep bruxism (SB), subject‐based awake bruxism (AB) including grinding, clenching, teeth contact, and bracing behaviours, along with measures of psychological distress (Patient Health Questionnaire‐4), perceived stress (Perceived Stress Scale), and resilient coping (Brief Resilient Coping Scale).

**Results:**

Arab participants reported the greatest psychological distress and perceived stress, yet they also displayed the strongest resilient coping. Regression analyses identified a distinct pattern in the Arab group regarding TMD and bruxism. Compared with a Jewish reference group, Arabs had markedly higher odds of TMD (OR = 2.58) and higher odds of grinding (OR = 1.81). In contrast, they showed lower odds for sleep bruxism (OR = 0.55) and awake bruxism behaviours of teeth contact (OR = 0.44) and bracing (OR = 0.55).

**Conclusion:**

The elevated TMD risk among Arab participants (more than double that of Jewish participants) suggests combined effects of chronic psychosocial stress and potential barriers to accessing health care. These findings highlight the need for culturally informed approaches to diagnosing and treating TMD and bruxism, particularly during periods of widespread societal stress.

## Graphical abstract

Wartime stress was associated with higher perceived stress and increased odds of TMD and bruxixm among Arab compared to Jewish participants.

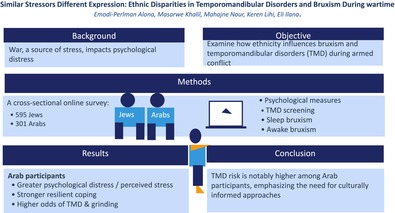

## 1 Background

Bruxism is a repetitive masticatory muscle activity characterized by clenching or grinding of the teeth, and/or bracing or thrusting of the mandible [1, 2]. Two forms of bruxism are acknowledged: Awake bruxism (AB) and Sleep bruxism (SB) [2]. The Standardized Tool for the Assessment of Bruxism (STAB) offers an effective biopsychosocial approach for identifying bruxism status, associated conditions, risk factors, and consequences [3]. Bruxism assessment utilizes three approaches: subject‐based (self‐report), clinically based, and device‐based. Despite self‐report's susceptibility to recall bias, it remains the most practical approach for epidemiological studies [1].

Psychosocial factors, such as stress and anxiety, were found to be associated with bruxism [4, 5, 6, 7, 8, 9]. Global stress situations such as the outbreak of the COVID‐19 pandemic, lead to aggravation of public psycho‐emotional status and result in bruxism symptoms intensification [10]. The adverse effects of the pandemic on SB and AB lasted longer and were more profound than initially assumed [11].

On October 7th, 2023, Israel faced an unprecedented terrorist attack. The sudden and unexpected attack posed an immediate physical and emotional threat to the entire society. The following urgent mobilization of the army reserves and the armed conflict that lasted 2 years increased anxiety, stress and anguish all around the country. The widespread psychological impact of the events was evidenced with approximately 60% of civilians outside the immediate conflict zones developing severe acute stress disorder [12]. Studies suggest a broad and significant impact of the events on the mental health of the Israeli population with a significant increase in post‐traumatic stress disorder (PTSD) and depression [13].

Israel represents a diverse, multi‐ethnic society, home primarily to two major population groups: Jews and Arabs. Although both groups are classified within the same racial category, their ethnic identities differ. Race and ethnicity describe distinct concepts [14]. Race relates to inherited physical and biological traits, whereas ethnicity encompasses shared cultural practices, historical experiences, traditions, beliefs, and social behaviours. Factors such as socioeconomic status, education, cultural norms, language use, and religious background can therefore shape individuals' psycho‐physiological responses to stress [15].

Studies examining the psychological impact of the recent events on civilians showed notable differences across groups. Relative to Jewish participants, Arab participants exhibited higher rates of probable PTSD, depression, and anxiety [16, 17]. Among adults living in the Gaza Strip the probability of experiencing psychological distress in 2025 increased twelvefold compared with levels reported 5 years earlier [18]. Laufer et al. indicated that after the terror attack, young Arab adults were more susceptible to secondary traumatic stress than Jewish peers, a pattern they linked to longstanding gaps in resources and ongoing sociopolitical strain [19].

### 1.1 Aim of Study

(i) To assess ethnic disparities in TMD, AB and SB among Jewish and Arab communities following the October 7th events; (ii) to evaluate the effect of stress perception, psychological distress and resilient coping on bruxism and TMD in the two populations.

Hypothesis:

(i) TMD and bruxism behaviour will differ between populations; (ii) severity of TMD, AB and SB will be affected by stress perception, resilient coping and emotional factors.

## 2 Methods

The study was planned as a cross‐sectional online survey using questionnaires. Distribution was carried out via social media platforms including WhatsApp, Facebook groups and Instagram. Each questionnaire commenced with a detailed explanation of the study's objectives, emphasized the anonymity of responses, clarified that the data would be used exclusively for research purposes and provided contact details of the research team. It further highlighted the voluntary nature of participation, noting that respondents could discontinue at any stage without repercussions. Participants could only access the survey once, after providing informed consent.

Data collection was conducted 1 year after the October 7th, 2023, attack, spanning November 2024 to September 2025.

## 3 Population

### 3.1 Jewish Population

A. An initial distribution of the questionnaires was conducted by one of the researchers (LK), who disseminated them both through social media platforms and via direct personal outreach to individuals within her immediate social environment. No compensation was offered. Data collection took place between November 14th, 2024, and September 25th, 2025, and resulted in 335 responses. The group was marked as *Jews‐A*.

B. As the initial sample size was deemed insufficient for the purposes of the study, supplementary recruitment was conducted through a professional survey company (Sekernet Ltd.), which specializes in online research and offers modest monetary compensation to panellists. Data collection took place between July 29th and July 31st, 2025, yielding a total of 260 responses. The group was marked as *Jews‐B*.

### 3.2 Arab Population

Distribution of the questionnaires was conducted by one of the researchers (KM), who disseminated them both through social media platforms and via direct personal outreach to individuals within his immediate social environment. Participation was uncompensated. Recruitment specifically targeted two Arab groups:

A. Arab individuals residing within the internationally recognized borders of Israel and holding Israeli citizenship. Data collection took place between November 9th, 2024, and September 29th, 2025, produced 237 responses and was classified under the label *Arab‐C*.

B. Arab individuals residing in the West Bank under the jurisdiction of the Palestinian Authority. Data collection took place between November 29th, 2024, and September 29th, 2025. A total of 64 responses were obtained, and the group was identified as *Arab‐D*.

The survey was presented in Hebrew to the Jewish population and in Arabic to the Arab population. The study was approved by Tel Aviv University's ethics committee (approval No. 0009166–3.).

Tools:

The following data were collected from participants:

### 3.3 Demographic and General Information

Consent to participate in study, age, gender, education and conjugal status (married/partnered, divorced/separated/widowed, single).

### 3.4 Temporo‐Mandibular Disorder (TMD) Screening

TMD screening was performed with the use of the 3Q/TMD questionnaire, a reliable and acceptable tool for screening TMD conditions in the general populations [20, 21]. It assesses whether individuals experience pain in the temple, face, or jaw during mouth opening or chewing at least once per week, and whether they encounter episodes of jaw locking with the same frequency. A positive response to one of the questions confirms the presence of TMD. The variable was coded as *TMD*.

### 3.5 Subject‐Based **SB** Assessment

Based on a question which refers to the presence of SB. The question originates from the Oral Behaviour Checklist (OBC) [22], is included in the Diagnostic Criteria for Temporomandibular Disorders (DC/TMD) [23] and has been adopted by the STAB (Axis A1, question A1.1) ^3^. Answer options were: 0‐ none of the time, 1‐less than one night/month, 2‐ one to three nights/month, 3‐one to three nights/week, 4‐ four to seven nights/week. A score of 2 or higher indicated positive awareness of the presence of SB behaviour. The variable was coded as *SB*.

### 3.6 Subject‐Based **AB** Assessment

Based on four questions referring to teeth grinding, teeth clenching, teeth contact and mandible bracing. The questions originate from the OBC [22], are included in the DC/TMD [23] and have been adopted by the STAB (Axis A2, questions A2.1‐A2.4) [3]. Answer options were: 0‐ none of the time, 1‐ little of the time, 2‐ some of the time, 3‐ most of the time, 4‐ all of the time. A score of 2 or higher on each item was interpreted as positive awareness of the corresponding AB behaviour. The variables were coded as: *AB‐grinding, AB‐clenching, AB‐teeth contact, AB‐bracing*.

### 3.7 Psychological Distress

Following the psychosocial assessment strategy of the DC/TMD [23] and recommended by the STAB (Axis B, Domain B1.1), the Patient Health Questionnaire‐4 (PHQ4) 3 was employed. PHQ4 is a brief screening tool, used for assessing anxiety and depression [24, 25]. Items are scored on a four‐point scale as follows: 0‐ not at all, 1‐ several days, 2‐ more than half the days; 3‐ nearly every day. The total score of the questionnaire ranges from 0 to 12, and the conditions are usually evaluated using the following cut‐off scores: 0–2, normal; 3–5, mild; 6–8, moderate; 9–12 severe psychological distress. The variable was coded as *PHQ4*.

### 3.8 Perceived Stress Scale

Perceived Stress Scale (PSS) is a most widely used psychological instrument for measuring the perception of stress [26, 27]. The instrument evaluates the frequency of stress‑related thoughts and feelings using a series of items rated on a Likert‑type scale, with higher scores indicating greater perceived stress. Score of 0–13 indicates low perceived stress; 14–26 indicates moderate perceived stress and of 27–40 indicates high perceived stress. The variable was coded as *PSS*.

### 3.9 Resilient Coping

Brief Resilient Coping Scale (BRCS) is a 4‐item questionnaire that captures tendencies to cope with stress adaptively and is currently recommended by the STAB (Axis B, Domain B1.2) [3]. The scale focuses on the tendency to effectively use coping strategies in flexible, committed ways to actively solve problems despite stressful circumstances [3, 28]. Answers are structured according to a 5‐point scale, rating from 1 (does not describe me at all) to 5 (describes me very well). Score of 4–13 points indicates low resilient coping; 14–16 medium resilient coping and 17–20 high resilient coping. The variable was coded as *BRCS*.

All questions were formulated in a first‐person voice (referring to self).

## 4 Statistics

Independent‐samples *t*‐tests, Pearson's chi‐square, and Kruskal‐Wallis tests compared variables between groups. Effect sizes were reported as Cohen's d (*t*‐tests), Cramér's V (chi‐square), and ε^2^ (Kruskal‐Wallis). Continuous variables were summarized using medians and interquartile ranges (IQR) and are presented as box‐and‐whisker plots showing the median, IQR, and whiskers extending to 1.5 × IQR.

Binary logistic regression was used to model binary outcomes. Ordinal outcomes were analysed using ordinal logistic regression models (PLUM procedure with a logit link). Regression coefficients were reported as log‐odds (B) with standard errors (SE), Wald χ^2^ statistics, and odds ratios (ORs) with 95% confidence intervals. The proportional odds assumption was assessed using the test of parallel lines. In cases where the proportional odds assumption was violated (*p* < 0.05), multinomial logistic regression models were fitted to examine associations between predictors and outcome categories.

## 5 Results

A total of 895 subjects participated in the survey (66.4% female).

### 5.1 Jewish Population

A total of 595 subjects responded to the questionnaire. 335 belonged to the Jews‐A group (mean age 37.47 ± 12.23) and 260 belonged to the Jews‐B group (mean age 43.65 ± 16.69). There were significant differences between the groups age‐wise, gender‐wise and education‐wise (*p* < 0.001 for all). Demographic characteristics of the Jewish groups are presented in Table 1.

**TABLE 1:** Demographic characteristics of the Jewish and Arab groups.

Group variable	Jews‐A (No, %)		Jews‐B (No, %)		Arab‐C (No, %)		Arab‐D (No, %)	
Gender ^a^								
Male	87	26%	129	46.6%	61	25.8%	24	37.5%
Female	248	74%	131	50.4%	175	74.2%	40	62.5%
Marital status								
Partnered	216	64.7%	164	63.1%	55	23.2%	12	18.8%
Previously married	16	4.8%	23	8.8%	3	1.3%	0	0
Single	102	30.5%	73	28.1%	179	75.5%	52	81.3%
Education								
Basic education	44	13.1%	84	32.3%	41	17.3%	13	20.3%
Partial academic education	48	14.3%	74	28.5%	57	24.1%	22	34.4%
Academic degree	243	72.5%	102	39.2%	139	58.6%	29	45.3%

^a^ One subject from the Arab‐C group did not specify gender. The total number of Arab participants was 237.

The Jews‐A group scored significantly higher on all psychological assessment variables (BRCS, PSS, PHQ4) and on self‑reported SB and AB behaviours (SB, AB‐grinding, AB‐clenching, AB‐teeth contact, AB‐bracing). Given the significant disparities identified between the Jews‐A and Jews‐B groups, across the collected measures, the groups were retained separately in subsequent analyses (see below).

### 5.2 Arab Population

A total of 301 Arab subjects responded to the questionnaire. 237 belonged to the Arab‐C group (mean age 27.46 ± 9.84) and 64 belonged to the in the Arab‐D group (mean age 25.72 ± 7.17). There were no significant differences between the two groups age‐wise and/or gender‐wise. Group Arab‐C was somewhat more educated than group Arab‐D (*p* < 0.05). Demographic characteristics of the two Arab groups are presented in Table 1.

With respect to psychological assessment variables (PSS, BRCS, PHQ4) and self‐reported oral behaviours (SB, AB‐grinding, AB‐clenching, AB‐teeth contact, AB‐bracing), the two Arab groups exhibited comparable profiles. Consequently, the Arab‐C and Arab‐D groups were aggregated into a single category, designated as *Arab‐total*, which was then used as one group in the comparison with the Jewish population (see below).

### 5.3 Comparisons Between Jewish and Arab Populations

#### 5.3.1 Psychological Measures

Significant differences were observed among the groups across all psychological measures (independent‑samples *t*‑test). For psychological distress (PHQ4), the Jews‐A group had a lower mean score (M = 4.33) compared with the Arab‐total group (M = 5.49, t_(634)_ = −4.95, *p* < 0.001, Cohen's d = −0.39). Similarly, the Jews‐B group scored lower (M = 3.55) than the Arab‐total group (t_(559)_ = −7.50, *p* < 0.001, d = −0.64). A significant difference was also found between the Jews‐A and Jews‐B groups (t_(540)_ = 2.88, *p* = 0.004, d = 0.24).

Perceived stress (PSS) scores followed a similar pattern. The Jews‐A group reported lower stress (M = 19.47) than the Arab‐total group (M = 20.06, t_(634)_ = −3.05, *p* = 0.002, d = −0.24), and the Jews‐B group scored lower (M = 18.24) than the Arab‐total group, (t_(559)_ = −4.92, *p* < 0.001, d = −0.42). The difference between Jews‐A and Jews‐B was also significant (t_(541)_ = 1.98, *p* = 0.048, d = 0.17).

Resilient coping (BRCS), was significantly lower in the Jews‐B group (M = 13.72) compared with the Arab‐total group (M = 14.98, t_(558)_ = −4.75, *p* < 0.001, d = −0.40), and in the Jews‐A group (M = 14.49) versus the Arab‐total group (t_(558)_ = −2.22, *p* = 0.027, d = −0.18). Additionally, Jews‐A scored higher than Jews‐B (t_(485)_ = 2.89, *p* = 0.004, d = 0.25).

### 5.4 TMD

Arab participants reported significantly higher rates of positive TMD complaints (76.4% among the Arab‐total group versus 28.5% and 51.6% in the Jew‐B and Jew‐A groups, respectively; Pearson Chi square, *p* < 0.001) with moderate to strong effect (Cramér's *V* = 0.38).

### 5.5 Bruxism Behaviours

**FIGURE 1:**
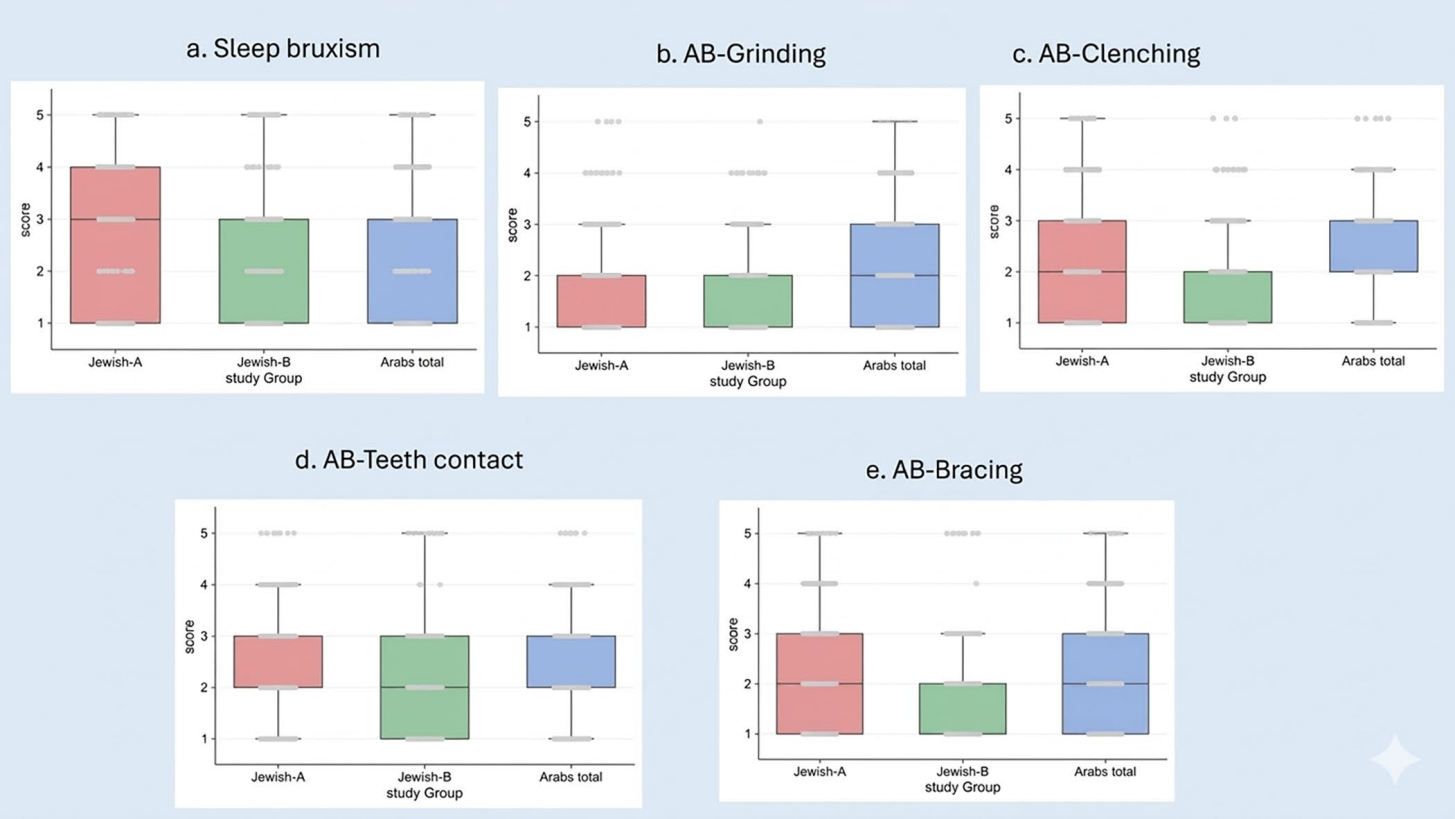
Boxplots showing the distribution of scores of SB and AB behaviours across three study groups. Boxes represent the interquartile range, lines indicate medians, and whiskers show values within 1.5 × IQR. Outliers appear as individual points.

There were significant differences among groups in SB, AB‐grinding, AB‐clenching, AB‐teeth contact and AB‐bracing behaviours (Kruskal‐Wallis, *p* < 0.001 for all) with medium effect (ε^2^ ranging 0.05 to 0.08). The Jews‑B group showed lower and more consistent bruxism behaviours. Jews‑A and Arab‐total groups showed higher medians and larger variability in several behaviours (Figure 1).

All results remained consistent when the analyses were conducted separately for men and women.

### 5.6 Modelling the Likelihood of TMD and Bruxism Behaviours Through Logistic Regression Analyses

Logistic regression analyses were performed to examine predictors of TMD and bruxism behaviours. All models included group (Jews‐A as reference, Jews‐B, Arab‐total), marital status, age, gender, education, PHQ4, PSS, and BRCS scores as predictors.

Ethnic group patterns revealed that Arab participants showed higher odds for TMD complaints (OR of 2.58) and AB‐Grinding (OR of 1.18) but lower odds for other behaviours, while Jews‐B consistently had lower odds than Jews‐A across all outcomes (ORs: 0.36–0.63).

Psychological distress (PHQ4) emerged as the strongest and most consistent predictor, significantly associated with increased odds across all outcomes (ORs: 1.135–1.24, all *p* < 0.001). Perceived stress (PSS) also showed consistent positive associations (ORs: 1.03–1.05).

Models explained 18%–27% of outcome variance (Nagelkerke R^2^ range: 0.18–0.27). Summary of Odds Ratios (OR) for Significant Predictors of TMD and Bruxism Behaviours is presented in Table 2. Complete model diagnostics are provided in supporting information (Tables S1–S3).

**TABLE 2:** Summary of odds ratios (OR) for significant predictors of TMD and bruxism behaviours.

Variable predictors	TMD	SB^a^	AB‐Grinding	AB‐clenching^a^	AB‐Teeth contact^a^	AB‐Bracing
Group						
Arab‐total versus Jews‐A	2.59	0.55	1.81	—	0.44	0.55
Jews‐B versus Jews A	0.41	0.36	0.63	0.37	0.40	0.54
Age	—	—	—	0.99	0.98	0.99
Gender (male)	—	—	1.37	—	—	—
PHQ4	1.13	1.17	1.14	1.18	1.24	1.22
PSS	1.04	1.04	1.05	1.04	1.03^b^	1.05
BRCS	1.05	—	—	—	1.05	—
**Model summary** (Negelkerke R^2^)	0.27	0.20	0.18	0.20	0.25	0.24

Abbreviations: AB, awake bruxism; BRCS, brief resilient coping scale; PHQ4, patient health questionnaire‐4; PSS, perceived stress scale; SB, sleep bruxism; TMD, temporomandibular disorders.

^a^ Proportional odds assumption violated.

^b^ Showed a trend toward statistical significance (*p* = 0.059).

## 6 Discussion

The recent turmoil in the Middle East has affected people from all communities, regardless of sex, religion, culture, or ethnicity. There was a significant increase in PTSD, anxiety, and depression [12], with some populations being disproportionately affected, mainly women and ethnic minorities [29].

Several studies documented the impact of the October 7th attack and the ensuing armed conflict on bruxism and TMD among the Jewish population. Jewish individuals exposed to the war conditions showed a marked increase in teeth clenching and exhibited a reduced ability to relax their masticatory muscles compared to subjects evaluated during a more peaceful period [30, 31]. Under the state of collective stress, anxiety and depression emerged as significant predictors of awake bruxism among Jewish subjects [31].

While knowledge about the effect of the regional war on the Jewish population is steadily increasing, data regarding the way the conflict affected bruxism and TMD in Arab communities are still limited. In the present study, Arab participants reported the highest levels of psychological distress and perceived stress, exceeding both Jewish groups, yet they also demonstrated the highest resilient coping. The pattern of distress aligns with previous findings [13, 16]. However, the elevated resilient coping observed among Arab participants diverges from the broader psychological resilience trends reported by Laufer et al. [19], who found that Arab participants demonstrated lower resilience than their Jewish counterparts. The discrepancy stems from the distinction between the two constructs: general resilience, as measured by Laufer et al. [19], reflects relatively stable internal traits such as persistence, confidence, adaptability, and emotional regulation, whereas resilient coping assessed in the current study captures the active strategies individuals use to manage stress, which can vary depending on the context.

The Arab population in Israel is a largely disadvantaged minority with major determinants of mental‐health problems and attitudinal barriers to mental health service [32]. More Arabs than Jews sought mental‑health support after the October 7th events, yet many encountered obstacles such as social stigma and limited access to care [16]. Communities exposed to chronic sociopolitical strain often develop strong coping repertoires, including collective identity, social cohesion, and culturally embedded support systems [33, 34]. Prior research among Arab communities in Israel has documented robust communal coping and meaning‐making processes that buffered the psychological impact of adversity. A study aimed to examine coping resources and stress reactions among Bedouin Arab adolescents in Israel underscored the importance of sense of coherence as a significant personal resource for coping with stressful political events [35].

Regression models revealed a distinctive pattern for the Arab group in relation to TMD and bruxism behaviours. Arabs showed substantially higher odds of TMD compared with the Jews‐A reference group (OR = 2.58) as well as significantly higher odds of AB‐Grinding (OR = 1.81). However, the same group exhibited lower odds across several bruxism behaviours, specifically sleep bruxism (SB; OR = 0.55), AB‐Teeth contact (OR = 0.44), and AB‐Bracing (OR = 0.55). The higher risk of TMD observed among Arab participants (over twice than among Jewish participants) might reflect the combined influence of chronic psychosocial stressors and potential obstacles to accessing health care. The finding that Arabs are 80% more likely to engage in AB‐grinding aligns with previous research which indicated that teeth grinding is a potential predictor of TMD‐related pain symptoms in a non‐patient population [36]. Given that the prevalence of TMD among Palestinian medical students exceeds 60%, [37] the elevated odds of TMD among Arab participants identified in the present study should be regarded as a significant public health concern for authorities in the region.

In addition to the disparities observed between Jewish and Arab participants, two further noteworthy findings emerged. First, the homogeneity of the two Arab groups. In spite political distinction, the two groups behaved in a similar way in almost all variables examined. This can be primarily attributed to deep‐seated common ancestral and familial roots, which appear to transcend current geopolitical boundaries. Historically, these two populations originate from the same extended families and clans that existed as single organic entities long before political partitions [38]. This genealogical bond creates a psychological continuity that may override the divergent legal contexts, reinforcing the notion of a ‘shared traumatic reality’ experienced by a singular, cohesive group rather than two distinct populations [39]. Despite living in different geographic contexts, a shared cultural basis may help explain the role of familial and cultural factors in shaping bruxism behaviours and TMD.

Second, the distinctions observed between the two Jewish groups may stem from differences in gender, age, and education, as well as from the contrasting data‑collection methods used, namely, recruitment through social media and personal outreach versus sampling through a professional survey company that compensates participants. Given that several studies examining the aftermath of the October 7th events relied on professional survey companies, [13, 16] this methodological factor should be considered when interpreting the findings.

### 6.1 Limitations

The groups evaluated in the present study are not necessarily representative of the Jewish and/or Arab population in Israel. Participants engaging through social media might have a specific interest in bruxism, while utilizing a pool of ‘professional responders’ could introduce different biases. Furthermore, the primary challenge in bruxism and TMD research is the difficulty of assessing it in large populations in a way that is both accessible and reliable. Although self‑report measures offer some diagnostic value, electromyography (EMG) remains the gold standard. In the present study, SB and AB behaviours were evaluated using subject based assessment, without objective confirmation through clinical and/or instrumental measures. Additionally, the psychosocial and behavioural variables examined were limited, and only a narrow range of associated pain conditions were considered. Future research should aim to deepen our understanding of TMD and bruxism behaviours by including larger and more diverse samples, incorporating a broader range of psychosocial factors, examining additional pain‐related conditions as well as a wider array of oral behaviours (e.g., the full OBC inventory).

## 7 Conclusions

The findings underscore the importance of a culturally sensitive perspective in diagnosing and managing TMD and bruxism, especially under times of collective stress. Clinicians working with Arab populations should consider psychosocial stress, emotional processing styles, and potential healthcare accessibility challenges when evaluating orofacial pain. Further research should explore how cultural norms, acculturation levels, and perceived discrimination contribute to TMD risk and modulate bruxism expression patterns in various populations.

## Author Contributions

Emodi‐Perlman Alona and Eli Ilana designed the research study; Masarwe Khalil and Keren Lihi were responsible for data collection, Masarwe Khalil and Mahajne Nour carried out the translation and adaptation of the relevant materials into Arabic. Emodi‐Perlman Alona and Eli Ilana analysed the data; Emodi‐Perlman Alona and Eli Ilana wrote the manuscript; All authors contributed to editorial changes in the manuscript. All authors read and approved of the final manuscript.

## Funding

This work was supported by a grant from the Israeli Science Foundatiom, No. 3737/24.

## Ethics Statement

The study was approved by the ethical committee of Tel‐Aviv University, approval No: 0008162–3. All participants signed an informed consent form.

## Conflicts of Interest

The authors declare no conflicts of interest.

## Supporting information


**Table S1:** Binary logistic regression predicting TMD complaints.
**Table S2:** Ordinal logistic regression predicting Sleep Bruxism frequency.
**Table S3:** Ordinal logistic regressions predicting AB‐behaviours frequencies.

## Data Availability

The data that support the findings of this study are available on request from the corresponding author. The data are not publicly available due to privacy or ethical restrictions.
