# Supplementary material for: Similar Stressors Different Expression: Ethnic Disparities in Temporomandibular Disorders and Bruxism During Wartime
Source: J Oral Rehabil. 2026 Apr 17;53(8):1509–16. doi: 10.1111/joor.70205 (PMC13358408; doi:10.1111/joor.70205)
Supplement: Supplementary file 1 — Table S1: Binary logistic regression predicting TMD complaints. Table S2: Ordinal logistic regression predicting Sleep Bruxism frequency. Table S3: Ordinal logistic regressions predicting AB‐behaviours frequencies. [file JOOR-53-1509-s001.docx]

Table S1: Binary logistic regression predicting TMD complaints

| **Predictor** | | **B** | **S.E.** | **Wald** | **df** | **P*** | **OR** | | **95% C.I. for OR** | | | |
| --- | --- | --- | --- | --- | --- | --- | --- | --- | --- | --- | --- | --- |
|  |  |  |  |  |  |  |  |  | Lower | | Upper | |
|  | Marital status: |  |  | 1.561 | 2 | 0.458 |  |  | |  | |  |
|  | Marital3(1) | -0.446 | 0.363 | 1.508 | 1 | 0.219 | 0.640 | 0.314 | | 1.304 | |  |
|  | Marital3(2) | 0.001 | 0.183 | 0.000 | 1 | 0.997 | 1.001 | 0.699 | | 1.433 | |  |
|  | Gender: male | 0.197 | 0.167 | 1.387 | 1 | 0.239 | 1.218 | 0.877 | | 1.690 | |  |
|  | Education: |  |  | 0.213 | 2 | 0.899 |  |  | |  | |  |
|  | Educ3(1) | 0.029 | 0.241 | 0.015 | 1 | 0.904 | 1.030 | 0.642 | | 1.651 | |  |
|  | Educ3(2) | 0.091 | 0.215 | 0.180 | 1 | 0.671 | 1.096 | 0.719 | | 1.669 | |  |
|  | **Group**: |  |  | 61.644 | 2 | 0.000 |  |  | |  | |  |
|  | **Jews B vs. Jews A** | -0.819 | 0.196 | 17.512 | 1 | **0.000** | **0.441** | 0.301 | | 0.647 | |  |
|  | **Arab-Total vs Jews-A** | 0.951 | 0.197 | 23.243 | 1 | **0.000** | **2.588** | 1.758 | | 3.810 | |  |
|  | **PHQ4** | 0.127 | 0.035 | 13.375 | 1 | **0.000** | **1.135** | 1.061 | | 1.215 | |  |
|  | **PSS** | 0.038 | 0.017 | 4.808 | 1 | **0.028** | **1.038** | 1.004 | | 1.074 | |  |
|  | **BRCS** | 0.053 | 0.027 | 3.991 | 1 | **0.046** | **1.055** | 1.001 | | 1.111 | |  |
|  | Constant | -2.162 | 0.540 | 16.021 | 1 | 0.000 | 0.115 |  | |  | |  |

Variables entered: Marital status, Gender, Education, Group, PHQ4, PSS, BRCS.

*Significant results marked in bold

† PHQ4- Patient health questionnaire-4; PSS- Perceived stress scale; BRCS- Brief resilient coping scale

Table S2. Ordinal logistic regression predicting Sleep Bruxism frequency

| **Predictor** | **B** | **SE** | **Wald χ²** | **OR** | **OR 95% CI** | | **P**** |
| --- | --- | --- | --- | --- | --- | --- | --- |
| **PHQ4** | 0.157 | 0.029 | 29.33 | **1.17** | 1.11 | 1.24 | **<.001** |
| **PSS** | 0.041 | 0.014 | 8.58 | **1.04** | 1.01 | 1.07 | **0.005** |
| BRCS | 0.035 | 0.022 | 2.53 | 1.04 | 0.99 | 1.08 | 0.11 |
| Age | -0.007 | 0.006 | 1.36 | 0.99 | 0.98 | 1 | 0.201 |
| **Group: Jewish-B vs Jewish-A** | -1.03 | 0.172 | 35.85 | **0.36** | 0.26 | 0.5 | **<.001** |
| **Group: Arab-total vs Jewish-A** | -0.598 | 0.161 | 13.79 | **0.55** | 0.4 | 0.75 | **<.001** |
| Gender: Male vs Female | -0.011 | 0.14 | 0.01 | 0.99 | 0.75 | 1.3 | 0.935 |
| In relationship: No vs Yes | 0 | 0.151 | 0 | 1 | 0.74 | 1.35 | 0.999 |
| Education: Some academic vs Low | 0.101 | 0.196 | 0.27 | 1.11 | 0.75 | 1.63 | 0.606 |
| Education: Academic degree vs Low | 0.229 | 0.177 | 1.67 | 1.26 | 0.89 | 1.78 | 0.197 |

*Reference categories were: Jewish-A (Group), Female (Gender), in a relationship = Yes, and Low education (Educ3). Analyses were conducted on complete cases for each model.

**Significant results marked in bold. Proportional odds assumption violated. Cumulative binary logistic regressions for Sleep Bruxism frequency, allowing predictor effects to vary across thresholds will be supplied upon request.

†PHQ4- Patient health questionnaire-4; PSS- Perceived stress scale; BRCS- Brief resilient coping scale

Table S3: Ordinal logistic regressions predicting AB-behaviors frequencies

| **Behavior** | **Predictor** | **B** | **SE** | **Wald χ²** | **OR** | **OR 95% CI** | | **P**** |
| --- | --- | --- | --- | --- | --- | --- | --- | --- |
| **AB-Grinding** | **PHQ4** | 0.133 | 0.031 | 18.41 | **1.14** | 1.08 | 1.21 | **<.001** |
|  | **PSS** | 0.046 | 0.016 | 8.27 | **1.05** | 1.02 | 1.08 | **0.003** |
|  | BRCS | -0.008 | 0.023 | 0.12 | 0.99 | 0.95 | 1.04 | 0.735 |
|  | Age | -0.003 | 0.006 | 0.25 | 1 | 0.99 | 1.01 | 0.587 |
|  | **Group: Jewish-B vs Jewish-A** | -0.467 | 0.187 | 6.23 | **0.63** | 0.43 | 0.91 | **0.013** |
|  | **Group: Arab-total vs Jewish-A** | 0.591 | 0.172 | 11.81 | **1.81** | 1.29 | 2.53 | **<.001** |
|  | **Gender: Male vs Female** | 0.311 | 0.153 | 4.13 | **1.37** | 1.01 | 1.84 | **0.042** |
|  | In relationship: No vs Yes | -0.07 | 0.167 | 0.18 | 0.93 | 0.67 | 1.29 | 0.674 |
|  | Education: Some academic vs Low | -0.099 | 0.21 | 0.22 | 0.91 | 0.6 | 1.37 | 0.638 |
|  | Education: Academic degree vs Low | 0.089 | 0.19 | 0.22 | 1.09 | 0.75 | 1.59 | 0.638 |
| **AB-Clenching^#^** | **PHQ4** | 0.166 | 0.029 | 32.72 | **1.18** | 1.12 | 1.25 | **<.001** |
|  | **PSS** | 0.036 | 0.015 | 5.76 | **1.04** | 1.01 | 1.07 | **0.015** |
|  | BRCS | 0.003 | 0.022 | 0.02 | 1 | 0.96 | 1.05 | 0.892 |
|  | **Age** | -0.013 | 0.006 | 4.69 | **0.99** | 0.98 | 1 | **0.022** |
|  | **Group: Jewish-B vs Jewish-A** | -1.004 | 0.176 | 32.49 | **0.37** | 0.26 | 0.52 | **<.001** |
|  | Group: Arab-total vs Jewish-A | -0.288 | 0.165 | 3.05 | 0.75 | 0.54 | 1.03 | 0.08 |
|  | Gender: Male vs Female | -0.136 | 0.144 | 0.89 | 0.87 | 0.66 | 1.16 | 0.343 |
|  | In relationship: No vs Yes | 0.009 | 0.155 | 0 | 1.01 | 0.74 | 1.37 | 0.954 |
|  | Education: Some academic vs Low | 0.243 | 0.199 | 1.49 | 1.28 | 0.86 | 1.88 | 0.222 |
|  | Education: Academic degree vs Low | 0.062 | 0.182 | 0.12 | 1.06 | 0.74 | 1.52 | 0.734 |
| **AB-Teeth Contact^#^** | **PHQ4** | 0.212 | 0.03 | 49.94 | **1.24** | 1.17 | | 1.31 |
|  | PSS | 0.028 | 0.015 | 3.49 | 1.03 | 1 | 1.06 | **<.001** |
|  | **BRCS** | 0.05 | 0.022 | 5.17 | **1.05** | 1.01 | 1.1 | 0.059 |
|  | **Age** | -0.018 | 0.006 | 9 | **0.98** | 0.97 | 0.99 | **0.023** |
|  | **Group: Jewish-B vs Jewish-A** | -0.912 | 0.174 | 27.44 | **0.4** | 0.29 | 0.56 | **0.001** |
|  | **Group: Arab-total vs Jewish-A** | -0.832 | 0.168 | 24.53 | **0.44** | 0.31 | 0.6 | **<.001** |
|  | Gender: Male vs Female | -0.013 | 0.144 | 0.01 | 0.99 | 0.74 | 1.31 | **<.001** |
|  | In relationship: No vs Yes | 0.217 | 0.154 | 1.99 | 1.24 | 0.92 | 1.68 | 0.926 |
|  | Education: Some academic vs Low | 0.041 | 0.199 | 0.04 | 1.04 | 0.7 | 1.54 | 0.16 |
|  | Education: Academic degree vs Low | 0.118 | 0.18 | 0.43 | 1.13 | 0.79 | 1.6 | 0.837 |
| **AB-Bracing** | **PHQ** | 0.195 | 0.03 | 42.25 | **1.22** | 1.15 | 1.29 | **<.001** |
|  | **PSS** | 0.044 | 0.015 | 8.6 | **1.05** | 1.02 | 1.08 | **0.003** |
|  | BRCS | 0.042 | 0.023 | 3.34 | 1.04 | 1 | 1.09 | 0.069 |
|  | **Age** | -0.012 | 0.006 | 4 | **0.99** | 0.98 | 1 | **0.037** |
|  | **Group: Jewish-B vs Jewish-A** | -0.624 | 0.181 | 11.88 | **0.54** | 0.38 | 0.76 | **<.001** |
|  | **Group: Arab-total vs Jewish-A** | -0.592 | 0.172 | 11.84 | **0.55** | 0.39 | 0.78 | **<.001** |
|  | Gender: Male vs Female | -0.204 | 0.152 | 1.8 | 0.82 | 0.6 | 1.1 | 0.181 |
|  | In relationship: No vs Yes | 0.241 | 0.16 | 2.27 | 1.27 | 0.93 | 1.74 | 0.132 |
|  | Education: Some academic vs Low | -0.054 | 0.208 | 0.07 | 0.95 | 0.63 | 1.42 | 0.795 |
|  | Education: Academic degree vs Low | 0.143 | 0.189 | 0.57 | 1.15 | 0.8 | 1.67 | 0.449 |

*Reference categories were: Jewish-A (Group), Female (Gender), in a relationship = Yes, and Low education. Analyses were conducted on complete cases for each model.

**Significant results marked in bold.

^#^ Proportional odds assumption violated. Cumulative binary logistic regressions for AB-Clenching and AB-Teeth Contact frequencies, allowing predictor effects to vary across thresholds, will be supplied upon request.

† PHQ4- Patient health questionnaire-4; PSS- Perceived stress scale; BRCS- Brief resilient coping scale
